# Enhanced Skin Performance of Emulgel vs. Cream as Systems for Topical Delivery of Herbal Actives (Immortelle Extract and Hemp Oil)

**DOI:** 10.3390/pharmaceutics13111919

**Published:** 2021-11-12

**Authors:** Vanja M. Tadić, Ana Žugić, Milica Martinović, Milica Stanković, Svetolik Maksimović, Almut Frank, Ivana Nešić

**Affiliations:** 1 Institute for Medicinal Plant Research “Dr. Josif Pancic”, Tadeusa Koscuska 1, 11000 Belgrade, Serbia; 2 Department of Pharmacy, Faculty of Medicine, University of Nis, Boulevard Dr. Zorana Djindjića 81, 18000 Niš, Serbia; milica.martinovic@medfak.ni.ac.rs (M.M.); milica.stankovic@medfak.ni.ac.rs (M.S.); ivana.nesic@medfak.ni.ac.rs (I.N.); 3 Faculty of Technology and Metallurgy, University of Belgrade, Karnegijeva 4, P.O. Box 3503, 11120 Belgrade, Serbia; smaksimovic@tmf.bg.ac.rs; 4 BAFA neu GmbH, Stephanstr. 2, D-76316 Malsch, Germany; almut.frank@bafa-gmbh.de

**Keywords:** immortelle, hemp oil, emulgel, cream, skin bioengineering

## Abstract

Immortelle, as rich source of chlorogenic acid and the phloroglucinol alpha-pyrone compound arzanol, possesses anti-inflammatory and antioxidant properties, affects cell regeneration, and has positive effect on many skin conditions. Hemp oil, characterized by a favorable omega-6 to omega-3 ratio, as well as an abundance of essential fatty acids and vitamin E, participates in immunoregulation and also act as an anti-inflammatory. In the present study, we examined the effect on the skin of creams and emulgels with immortelle extract and hemp oil, by comparing them to placebo samples and a non-treated control. A long-term in vivo study of biophysical skin characteristics, which lasted for 30 days, was conducted on 25 healthy human volunteers. Measured parameters were electrical capacitance of the *stratum corneum*, trans-epidermal water loss (TEWL), and skin pH and erythema index. Further, a sensory study was carried out in which the panelists had to choose descriptive terms for sensory attributes in questionnaire. The results showed that application of all preparations led to increase of skin hydration and TEWL reduction, while the skin was not irritated, and its normal pH was not disrupted. This study also showed importance of the carrier. Not only were emulgels described by panelists as preparations with better sensory properties, there was a significant difference between the skin hydration effect of emulgel with immortelle extract and hemp oil compared to the placebo emulgel, which was not the case with creams. Such findings indicated enhanced delivery of herbal active substances from emulgel compared to the cream.

## 1. Introduction

The *Helichrysum italicum* (Roth) G. Don fil. subsp. *italicum* (known as immortelle) plant, belonging to the family Asteraceae, is a shrub which is used in phytomedicine throughout the Europe, particularly in Mediterranean countries. Extracts of the aerial parts of this plant are used as antimicrobial, anti-inflammatory, antioxidant, and anti-allergen agents, for the treatment of respiratory diseases and different skin disorders such as eczema, psoriasis, and inflammatory conditions of the skin, as well as for protection against lipid peroxidation caused by oxidative stress [1,2,3,4]. Extracts of *H. italicum* are used in cosmetic industry, as ingredients of fragrances or in preparations for the protection of sensitive and irritated skin. It is considered that beneficial effects on the skin are due to the high flavonoid, coumarin, phenolic acid, acetophenone, and α-pyrone derivative content in the plant [5]. As a matter of fact, during the last decade, significant attention has been paid on the activity of arzanol, phloroglucinol α-pyrone, for the first time isolated from the acetonic extract of the species *H. italicum* subsp. *microphyllum*, characteristic of the Sardinian region. Arzanol exhibited significant anti-inflammatory activity against carrageenan-induced pleurisy of rats, and inhibited the biosynthesis of pro-inflammatory eicosanoids [6]. In the work of Appendino et al., 2007, it was shown that arzanol might take a part in blocking the NF-kB activation, along with helypyrone and the release of several pro-inflammatory mediators in isolated human monocytes, as well as stopping HIV-1 replication in T-cells. Arzanol has also been reported to possess significant antioxidant activity in various in vitro systems of lipid peroxidation [7,8]. On the other hand, phenolic acids, and especially depside acids, such as chlorogenic acid, possess potent antioxidant properties by increasing the activity of superoxide dismutase, catalase, and glutathione, and decreasing lipid peroxidation. Recently, investigation of topical application of chlorogenic acid revealed that it can accelerate the process of excision wound healing through its antioxidant potential, and its significant ability to increase collagen synthesis through upregulation of key players in different phases of the wound healing process [9].

On the other hand, industrial hemp oil isolated from plant *Cannabis sativa* L. (family Cannabinaceae), which is used in agriculture, has high nutritional and health-beneficial value. Due to its high level of proteins, carbohydrates, oils, minerals, and fibers, hemp seeds reduce cholesterol and high blood pressure, and have long been used in traditional medicine and cosmetics for the preparation of detergents, soaps, body oils, and creams [10]. Previous studies indicated the possibility of using the hemp oil as ingredient in skin preparations, such as body oils and creams. The emphasis is placed on the possible application of hemp oil for dry skin conditions, due to its balanced content of polyunsaturated fatty acids, and a unique ratio of essential fatty acids. For this reason, hemp oil could be beneficial for the treatment of different skin diseases, such as eczema, dermatitis, psoriasis, lichen planus, and acne rosacea. This oil also improves skin immunity against bacterial and viral infections, as well as fungal infections [10,11].

Vehicles that are used for incorporation of the active substances are of great importance when it comes to substance release and dermal delivery [12,13]. In addition, the selection of proper formulation of the vehicle is further related to the sensory properties of the final topical preparation, often decisive for consumer acceptance i.e., patient compliance, aside from being the largest feature of the products’ sales potential [14]. Emulsion-based formulations are often used for topical delivery of active substances, since they can establish good interaction with lipids and water in the *stratum corneum* [15]. Therefore, creams as emulsion-based formulations are very popular–especially O/W creams that are easily applied and spread, and leave light feeling on the skin [12]. Emulgels, as emulsions whose outer phase is gelled with the appropriate gelling agents, represent dual control/release systems, due to the existence of gel’s network as a structure that provides emulsion stabilization alongside additional control in drug release [16]. Since emulgels have characteristics of both emulsions and gels, they are well accepted with patients [17,18].

The aim of the study was to investigate the effects on the skin of creams and emulgels with immortelle extract and hemp oil in an in vivo study conducted on healthy human volunteers. Furthermore, the goal was to compare the influence of the carrier (creams versus emulgels) on the effect of the tested formulations containing the same active ingredients (immortelle extract and hemp oil) on the skin. To additionally compare the two carriers, evaluation of their sensory characteristics was also assessed.

## 2. Materials and Methods

Mixed emulsifier (glyceryl stearate, ceteareth-20, ceteareth-12, cetearyl alcohol, and cetyl palmitate), polysorbate 80, and glycerin were purchased from Sabo (Italy). Cetyl alcohol, stearyl alcohol, and butyrospermum parkii were obtained from Cognis (Monheim, Germany), while carbomer was bought from Lubrizol (Ovele Westerlo, Belgium). Tocopheryl acetate and BHT were procured from Merck (Darmstadt, Germany). Caprylic/capric triglycerides were acquired from Henkel (Düsseldorf, Germany), sodium benzoate and sodium hydroxide from Zdravlje (Belgrade, Serbia), propylene glycol from Fagron Hellas (Trikala, Greece), and distilled water from a Medical Faculty in Niš (Serbia). All chemicals used were of analytical purity (p.a.), or HPLC grade (ACN; Fisher Scientific UK Ltd., Loughborough, UK), chlorogenic acid and arzanol (purity for both ≥98%, Sigma, St. Louis, MO, USA). Hemp oil was obtained using the hydraulic pressing of shelled industrial hemp seeds *(Cannabis sativa* L., Cannabinaceae), produced by Bafa European Hemp Foods (Stephanstr. 2, DE-76,316 Malsch, Germany), while immortelle extract was prepared from *Helichrysum italicum* (Roth) G. Don fil. subsp. *italicum*, Asteraceae, using a supercritical carbon dioxide extraction (SFE) procedure. The plant was cultivated in the region of Konavle (the southern part of Croatia) and the voucher specimen was deposited at Institute for Medicinal Plant Research “Dr. Josif Pančić“, Belgrade, Serbia. SFE was carried out in a high-pressure extraction-adsorption unit (HPEA 500, Eurotechnica GmbH, Bargteheide, Germany). The SFE was performed at 350 bar and 40 °C. Approximately 15 g of milled flowers were placed in a 280 mL stainless steel extractor (internal diameter and height 38 mm and 254 mm, respectively), designed to be operated at a maximum pressure of 550 bar and temperature of 120 °C. Raw plant material was first put in a metal tube with an internal diameter and height of 30 mm and 240 mm, respectively, closed at the perforated bottom with filter paper, and then subjected to the extractor vessel. After the desired temperature in the extractor was achieved, the system was pressurized. Liquid CO_2_ supplied from a CO_2_ cylinder with a siphon tube was cooled in a cryostat to prevent vaporization, and pumped into the system by a liquid metering pump (Milton Roy, Pont Saint Pierre, France). After reaching the operating conditions, the continuous flow of supercritical fluid commenced. The operating pressure was maintained by the back pressure regulator (BPR). The extraction time was 3.5 h.

### 2.1. High-Performance Liquid Chromatography (HPLC) Analysis of SCO_2_ Immortelle Extract

“Fingerprinting” of immortelle extract was achieved using an Agilent Technologies 1200 HPLC (Santa Clara, CA, USA), equipped with a Lichrospher 100RP 18e column (150 × 3.2 mm, 5 μm, Agilent Technologies, Santa Clara, CA, USA), applying a gradient elution of two mobile phases, i.e., “A/B” (“A”-0.2 M solution of phosphoric acid, and “B”-being a pure acetonitrile) at flow–rates of 1 mL/min, with photodiode-array (PDA) detection (UV at 280 nm), always within 100 min. Winning combinations were 89–75% A (0–35 min); 75–60% A (35–55 min); 60% A, isocratic for 10 min, 60–50% A (65–70 min), at 50% A, isocratic for 5 min, and 50–43% A (75–78 min), at 43% A, isocratic for 5 min, 43–23% A (83–85 min); at 23% A, isocratic for 5 min, 23–11% A (90–95 min); at 11% isocratic for 5 min. The concentration of investigated sample was 21.50 mg/mL. Before injection, sample was filtered through a PTFE membrane filter. For standards used in the investigation, the concentrations were 0.21 mg/mL for chlorogenic acid, and 0.67 mg/mL for arzanol. The volume of standard solutions being injected, as well as for the tested sample extract, was 4 µL. The identification was based on retention time and spectra matching with those of standards. Once spectra matching succeeded, results were confirmed by comparison with a respective standard to achieve a complete identification by means of the so-called peak purity test.

### 2.2. GC and GC/MS Analysis of Fatty Acids in Hemp Oil

The sample of hemp oil was analysed after converting the fatty acids to methyl esters using AOAC procedure 965.49 [19] with some modifications. An extract (20.55 mg) was mixed with a methanol-sulphuric acid mixture. The mixture was carefully heated for 2 h under reflux to enable the moderate boiling. Once reaction was complete, the cooled mixture was washed twice with 50 mL of petroleum ether, and once again with 20 mL of distilled water. The collected petrol ether fraction was dried using the anhydrous Na_2_SO_4_, and the solvent was evaporated on a vacuum evaporator. The chemical composition of the fatty oil was analysed using the gas chromatography with flame ionization detector (GC/FID) and gas chromatography-mass spectrometry (GC/MS) techniques. GC analyses were performed on a Shimadzu GCMSQP2010 ultra mass spectrometer (Kyoto, Japan) fitted with a flame ionic detector, and coupled with a GC2010 gas chromatograph. The InertCap5 capillary column (60.0 m × 0.25 mm × 0.25 µm) was used for separation. Helium at a split ratio of 1:5 and a linear velocity of 35.2 cm/s was used as a carrier gas. The ion source temperature was 200 °C, the injector temperature was 250 °C, and the detector temperature was 300 °C, while the column temperature was linearly programmed from 40 to 260 °C (at rate of 4 °C/min), from 260 to 310 °C (at rate 10 °C/min), and, after reaching 310 °C, kept isothermally for 10 min. The derivatized samples were dissolved in hexane, and consecutively injected in amount of 1 µL. The content of different compounds was determined on the basis of area of chromatograms, and defined as content according to the GC area. The identification of the constituents was performed by comparing their mass spectra and retention indices (RIs) with those obtained from authentic samples and/or those listed in the NIST/Wiley mass-spectra libraries, using different types of searches (PBM/NIST/AMDIS) and available literature data [20].

### 2.3. Preparation of Investigated Cream Samples

Samples were prepared by adding heated aqueous phase (75 °C) to the heated oil phase (70 °C) (Table 1) on thermostatic heating plate of magnetic stirrer IKA-MAG (IKA Werke, Staufen, Germany). A propeller rotary laboratory stirrer RW16 basic (IKA Werke) was used for stirring. At a temperature of 40 °C, herbal active substances (hemp oil and immortelle extract) were added into active cream sample (AC), and the stirring was continued until the preparation had cooled. The placebo cream (PAC) sample did contained neither hemp oil nor immortelle extract.

### 2.4. Preparation of Invetigated Emulgel Samples

Sodium benzoate was dissolved in a glass vessel in a mixture of propylene glycol and water. Carbopol Ultrez 10 was added gradually over the surface. The heated aqueous phase at 75 °C (Table 2) was added gradually to the heated oil phase (70 °C), with stirring using a propeller rotary laboratory stirrer RW16 basic (IKA Werke) at 500 rpm. At an emulgel temperature of 40 °C, a 10% solution of sodium hydroxide was added to the formulation, and, after vigorous mixing, the active components (hemp oil and immortelle extract) were added. Stirring was continued until the emulgel (EM) had cooled and reached room temperature. Placebo emulgel (PEM) was prepared in the same manner, with the exception that the active substances were not added.

### 2.5. Skin Study Design

#### 2.5.1. In Vivo Test Protocol

The testing of the effects of creams and emulgels on the skin was conducted using in vivo non-invasive biophysical techniques. For this purpose, the device Multi Probe Adapter MPA^®^9 (Courage&Khazaka Electronic GmbH, Köln, Germany) with different probes, previously calibrated, was used. The probe Corneometer^®^CM 825 was used for measuring electrical capacitance (EC) which depicts stratum corneum hydration, while the probe Tewameter^®^TM 300 was used as a measuring device for the assessment of trans-epidermal water loss (TEWL). The probe skin-pH-Meter PH 905 was used as quick tool for measuring the pH of the skin, whilst the erythema index (EI) was measured using a Mexameter^®^MX.

The study lasted for 30 days. The participants who took part in it were 25 healthy volunteers (mean age 23.36 ± 0.64 years) of both genders (20 women and 5 men), who had no past or present history of skin diseases, nor had they used systemic or topical drugs within two weeks prior to the study. All volunteers were informed about the study protocol, and signed the written informed consent form. The research was conducted in accordance with the Helsinki Declaration, and permitted by the Ethics Committee of the Medical Faculty in Niš (Serbia), protocol code 12-6316-2/8 from 16 June 2016. The entire study was carried out in consonance with the guidelines and published recommendations.

The specific areas sized 9 cm^2^ on the volar part of right forearms of volunteers were determined for treating with emulgels (active–EM and placebo–PEM), while left forearms were reserved for creams (active–AC and placebo–PAC) application. This type of site assignation was used in order to compare the effects of active samples with the corresponding placeboes, i.e., for a better understanding of the possible effects of herbal active ingredients on the human skin. In addition, on the left arm, one area was defined as a non-treated control (NC) that was not treated with any of the tested samples. All participants were given preparation samples marked with place of application.

The day before the start of the study, basal values of all biophysical parameters were measured at all designated areas (on the volar parts of forearms). The participants were told to apply the tested samples on defined areas two times a day, (morning and evening) for 28 days. Moreover, they were instructed not to apply any other product on the test-sites during the study. The following measurements were performed after 7, 14, and 28 days of application in the morning, before applying the sample preparations. The last measurement took place 2 days after cessation of application of the samples. Each measurement was conducted under precisely defined conditions to which the volunteers were subjected before measurement (30 min rest with uncovered forearms, at room temperature 21 ± 2 °C, and relative humidity 45 ± 3%).

#### 2.5.2. Examination of Sensory Properties

In order to assess certain sensory characteristics of the investigated cream and emulgel samples, 25 human volunteers were given a questionnaire (Table 3) in which the sensory descriptors were listed. They estimated characteristics of samples before rubbing, and during spreading, as well as the feeling after application. All panelists had to choose descriptive term for each sensory attribute listed in questionnaire. The sensory evaluation was carried out in the properly illuminated laboratory, at room temperature 21 ± 2 °C, and under relative humidity 45 ± 3%.

### 2.6. Statistical Analysis

The results of in vivo measurements were presented as mean ± standard error for each parameter. Measured values were compared to basal values, and changes in values during the study were analyzed. Further, samples with herbal active principles were compared to placebo samples and the non-treated control. Moreover, active emulgel was compared to the active cream in order to examine influence of the carrier on the effects of the final preparation on the skin. The statistical analysis (Student’s *t*-test and ANOVA) was performed using IBM SPSS Statistics 20 (IBM Corporation, Tokyo, Japan).

## 3. Results

**Figure 1:**
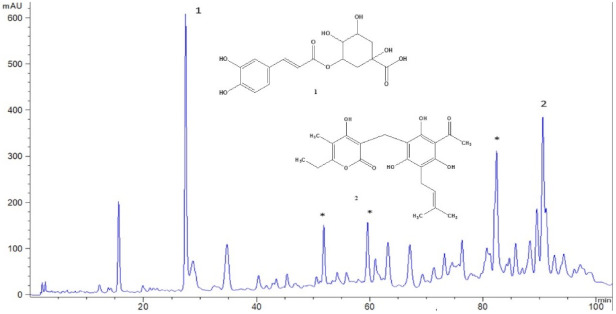
HPLC chromatogram of the immortelle SCO_2_ extract used in the experiment, recorded at 280 nm: chlorogenic acid (1) and arzanol (2). *—phloroglucinol derivatives (gamma-pyrone derivatives).

Figure 1 represents the fingerprint of used immortelle SCO_2_ extract. Chlorogenic acid (1) and phloroglucinol derivatives, among them arzanol (2), gave the most prominent peaks. Their contents were determined to be 11.51% and 4.59% for chlorogenic acid and arzanol, respectively. The chemical profile of the used hemp oil is given in Table 4.

The effects of the investigated samples of active cream with immortelle extract and hemp oil (AC) and active emulgel with the same active components (EM) on the skin parameters of healthy volunteers were investigated in relation to the appropriate placebo formulations (PAC and PEM) and appropriate control, i.e., non-treated skin site (NC). The active samples were also compared with each other, regarding to the applied carrier (cream or emulgel) used for the incorporation of the mentioned herbal actives.

**Figure 2:**
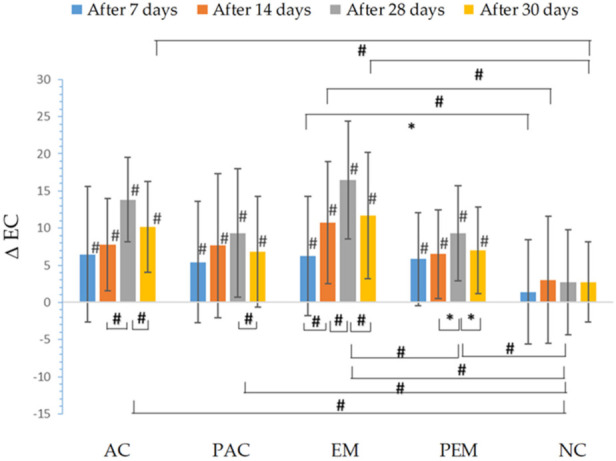
In vivo determined absolute changes relative to basal values of electrical capacitance (EC) for investigated creams (active cream—AC, placebo cream—PAC) and emulgels (active emulgel—EM, placebo emulgel—PEM) after 7, 14, and 28 days of application, and 2 days after cessation of application. Significant differences are marked with # (*p* < 0.01) and * (*p* < 0.05).

The changes of electrical capacitance (EC) indicated a moisturizing effect of the tested cream and emulgel (Figure 2). All samples showed an increase in skin hydration throughout the study, which was statistically significant, compared to the corresponding basal values. EM significantly increased the hydration of stratum corneum after seven days of use, compared to the initially assessed values, as well as compared to NC test-site. This trend was also observed in measurements after 14 and 28 days of EM skin treatment (after 28 days of application, we noticed a statistically significant increase in skin hydration compared to the PEM, as well as the NC). Moreover, the increase in stratum corneum hydration after treatment with emulgel between the 7th and 14th day, as well as between days 14 and 28, was also statistically significant. On the other hand, the use of PEM led to an increase in stratum corneum hydration, which was statistically significant compared to the initially measured values (before treatment), but not to the NC (with the exception of the measurements after 28 days of application). Taking this finding into account, it may be assumed that the effect of the significant increase in hydration of the stratum corneum is related to the presence of active compounds (hemp oil and immortelle extract). On the other hand, this cannot be claimed for the sample AC which contained the same active ingredients in the same concentrations. Notably, although this sample led to a significant increase in stratum corneum hydration compared to the initial measurements, this increase was significant only after 28 days of its usage, in comparison to the NC. There was an increase in stratum corneum hydration after treatment with AC, compared to its placebo, but with no statistically significant difference. However, PAC showed a statistically significant increase in stratum corneum hydration after 28 days of treatment, compared to NC, suggesting that the revealed effects of this sample may be predominantly attributed to the vehicle itself. Two days after cessation of use (day 30), there was a significant decrease in hydration, compared to the 28th day of measurement at all test sites. However, in the case of both EM and AC, this value was still significantly higher than the one measured at the non-treated control test-site, which cannot be stated for sites treated with placebo samples (PEM and PAC).

**Figure 3:**
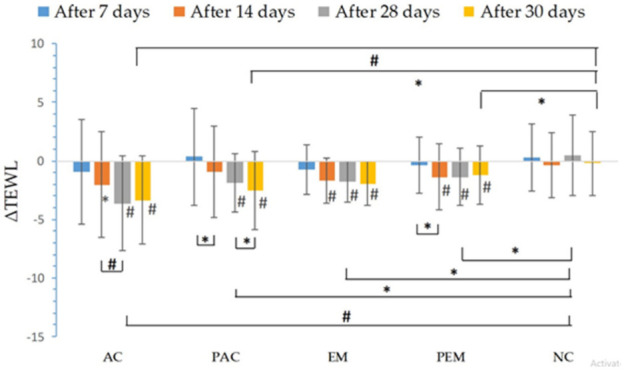
In vivo determined absolute changes relative to basal values of trans-epidermal water loss (TEWL) for investigated creams (active cream—AC, placebo cream—PAC) and emulgels (active emulgel—EM, placebo emulgel—PEM) after 7, 14, and 28 days of application, and 2 days after cessation of application. Significant differences are marked with # (*p* < 0.01) and * (*p* < 0.05).

After four weeks of sample application on the skin, all of them led to a significant reduction of TEWL compared to the NC test-site, where none of the tested samples were applied (Figure 3). TEWL values were reduced statistically significant relative to the corresponding basal values after application of all samples. There was no statistically significant difference in the TEWL values between the active samples, as well as between active versus corresponding placebo samples. After 28 days of application of all samples, a statistically significant decrease of TEWL values was noticed compared to the NC site.

**Figure 4:**
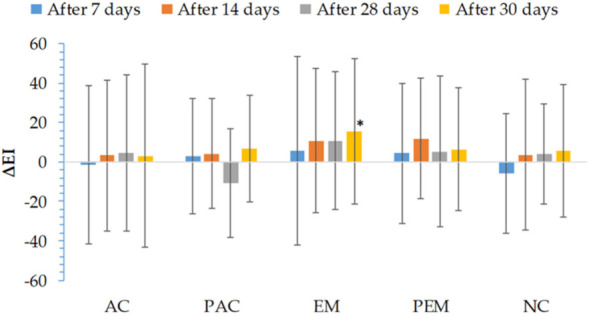
In vivo determined absolute changes relative to basal values of erythema index (EI) for investigated creams (active cream—AC, placebo cream—PAC) and emulgels (active emulgel—EM, placebo emulgel—PEM) after 7, 14, and 28 days of application, and 2 days after cessation of application. Significant differences are marked with * (*p* < 0.05).

During the study, there were no statistically significant changes in erythema index (EI) after application of the tested samples. None of the applied samples caused the appearance of any form of skin irritation or redness. Only in the case of the EM test-site was the EI value was significantly higher (2 days after cessation of EM application), compared to the basal value. During the long-term application of the samples for 28 days, there were no significant changes in the values of this parameter. There was no statistically significant difference in the values of this parameter between the applied samples, as well as compared to the non-treated control. Overall, it may be stated that EI measurements indicated good tolerability of all tested formulations on the skin (Figure 4).

**Figure 5:**
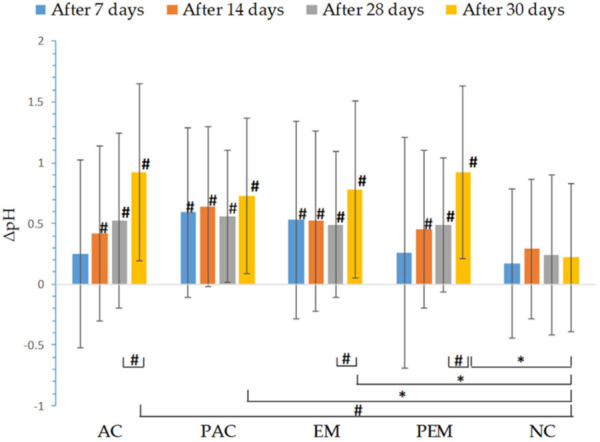
In vivo determined absolute changes relative to basal values of pH for investigated creams (active cream—AC, placebo cream—PAC) and emulgels (active emulgel—EM, placebo emulgel—PEM) after 7, 14, and 28 days of application, and 2 days after cessation of application. Significant differences are marked with # (*p* < 0.01) and * (*p* < 0.05).

From the 14th day of measurement, the increase in skin pH after treatment with all samples was statistically significant, compared to the initially measured values. There were no statistically significant differences in the values of this parameter between the active samples (the emulgel and the cream) and their respective placebos. In comparison with the NC test-site, pH changes were statistically significant only on the 30th day after the initial measurement (the 2nd day after discontinuation of treatment) for all tested samples (Figure 5). However, the measured pH values did not exceed normal physiological skin pH values (4–6) during the whole experiment [21].

Sensory analysis of the investigated creams showed that all of the samples were characterized by panelists as easy to spread, not sticky, and not greasy during application (Table 5). All of them caused a slight shine of the skin after application and left moderate film, but did not leave a greasy feeling behind. Both creams were described as semisolid by majority of panelists (over 84%), while less than 70% of the panelists described emulgels as semisolid. Expectedly, creams were labeled as thicker than the emulgels. Therefore, emulgels were described to have lighter structure than creams, which is an important factor when considering the aspect of compliance of the patients. Moreover, 100% of the panelists stated that skin after EM application was not greasy nor sticky, which was not the case for other preparations. Emulgel samples (EM and PEM) were described to have a lower rate of absorption, compared to the creams.

## 4. Discussion

The skin, composed of several layers, represents an effective barrier against external influences and the penetration of other substances through it. Nevertheless, it is very popular way of administering the active substances, especially when it comes to treatment of local cutaneous problems [22]. Delivery of active substance itself is also influenced by its physical and chemical properties (solubility, stability, bioavailability etc.), as well as by the delivery vehicle [23]. Proper choice of delivery vehicle is of great significance, therefore many delivery systems are being developed, of which the most popular are emulsion-based vehicles (creams, lotions, micro-emulsions, nano-emulsions etc.) and gels (hydrogels etc.) [24,25]. Emulsion, composed of two phases, acts as a controlled release system, in which a substance is stored in internal phase, and is released in external phase, and absorbed afterwards [26]. Improved emulsion-based delivery systems are nano-emulsions, in which the diameter of the dispersed droplets is 20–200 nm. These systems offer increased bioavailability of drugs, and better permeation through the skin [27]. Gels, on the other hand, are characterized by a network structure in which it is possible to capture large amounts of water that consequently lead to better dissolution of the active substance, which is mainly hydrophilic [25]. In addition, the migration of the substance to the skin is much easier than with emulsions, which leads to the famous immediate cooling and hydration effect of gels [26,28]. However, when it comes to the delivery of hydrophobic substances and interactions with the hydro-lipid layers of the skin, emulsions (lotions and creams) have a dominance over gels [15]. Emulgels, formed as dual delivery systems which represent the combination of emulsions and gels, can therefore be given the advantages of both of them, such as thixotropy, good spreadability, and better patient acceptability, greaseless, emollient, better stability of the active substance (especially hydrophobic ones), and better stability of the preparation itself [16]. Compared to nano-vesicular structures such as liposomes and niosomes, emulgels offer better loading capacity due to networks formed by a gelling agent, and are also easier to prepare, since their production does not require special equipment [17]. The benefits of releasing active substances from emulgels have been investigated in many studies, which has led to the wide application of emulgels in the medical and cosmetic field [29,30,31,32,33,34,35].

In this in vivo study conducted on healthy human volunteers, we examined the effects on the skin of creams and emulgels with immortelle extract and hemp oil. Moisturizing effect of preparations was evaluated by measuring electrical capacitance of stratum corneum as a measure of its hydration; skin barrier function correlated with changes in TEWL, tolerability of preparations was shown by changes of EI, and influence of the preparation on the pH of the skin was acquired by the obtained pH values. Small standard deviations in the values of certain tested parameters may be explained, among the other things, by the narrow age range of the volunteers who participated in the study.

Both immortelle extract and hemp oil showed positive effects on skin in previous studies. For instance, night cream containing immortelle extract showed improvement in skin hydration by 64.4% after 1h of application, and reduced TEWL by 10% for the same time in the study conducted on 117 volunteers [36]. Hemp seed oil significantly reduced TEWL values, as well as subjective feelings of dryness and itchiness in group of volunteers with atopic dermatitis after eight weeks of usage [37].

Detailed literature survey indicated immortelle extract to be a rich source of natural bioactive compounds that possess anti-inflammatory, antioxidant, and also antimicrobial activity against Gram+ bacteria, and may have positive effects on cell regeneration, acnes, and certain skin conditions such as dermatitis, eczema, psoriasis, rosacea, scars, ulcers, and wounds. The stated effects of immortelle extract are usually attributed to phenolic and flavonoid compounds [1,4,38,39]. Indeed, HPLC analysis of the immortelle extract used in our study revealed that it was rich in chlorogenic acid (approximately 11.5%) and arzanol (4.59%), confirming previous findings. Chlorogenic acid is a well-known antioxidant, and polyphenol plays a role in skin cancer prevention and skin-photoprotection [40]. Arzanol inhibits biosynthesis of pro-inflamatory eicosanoid mediator, and therefore acts as an anti-inflammatory in vivo [6].

Hemp seed oil, rich in essential fatty acids, have a favorable omega-6 (*n*-6) to omega-3 (*n*-3) ratio 2:1–3:1 [41]. The results of GC and GC/MS analysis of industrial hemp oil used in tested preparations showed that the oil has a defined content of polyunsaturated fatty acids (PUFA), in which the ratio of linoleic (LA) and linolenic (LNA) acid is 3:1 (optimal ratio of these ω-3 and ω-6 acid) without tetrahydroocannabinol, as well as the presence of vitamin E. It is known that PUFA participates in immunoregulation, and has beneficial effect on inflammatory skin conditions, as well as on the TEWL index and skin barrier [42,43].

Dry, inflexible, and damaged skin is not just an aesthetic problem. The stratum corneum needs an appropriate amount of water in order to fulfill its barrier function. Moreover, proliferation and differentiation of the epidermal cells cannot be well regulated without adequate stratum corneum hydration. Moisturizing preparations have shown very positive effects on the skin, especially dry skin [44]. In our study, it was shown that immortelle extract and hemp oil may influence moisture of the skin. There was statistically significant difference between the hydration of stratum corneum after 28 days of administration of emulgel and cream containing these herbal drugs in comparison with basal values.

Not only did EM application lead to significant increase in electrical capacitance of the skin compared to the non-treated control, there was also a significant difference between increase of stratum corneum hydration measured at the test-site treated with EM, compared to the one treated with PEM, which was not the case with AC and PC (Figure 2). This indicates the importance of proper carrier selection. Probably, due to its “dual” structure (emulsion + gel), emulgel offers the possibility of enhanced release of the active substance (first of all, chlorogenic acid and arzanol, determined in *H. italicum* scCO_2_ extract, as well as PUFA quantified in the hemp seed oil, but not excluding the influence of other components present in lower concentration). A similar effect was previously observed in a study with clotrimazole, in which emulgel enabled increased release of the active substance compared to the cream [45]. Moreover, the rate release constant of miconazole-nitrate from emulgel was greater than the one from the cream [46].

TEWL is a parameter that indicates the permeability and integrity of the skin. Hence, low TEWL values are linked with healthy and normal skin, while increased values indicate damaged skin [47,48,49,50]. Application of all of the tested preparations (creams and emulgels) led to a decrease in TEWL, and therefore led to improvement of the skin barrier. Further, the majority of study participants described that all samples left a moderate film on the skin after application, which can also influence TEWL.

Application of the investigated samples did not cause unacceptable pH values of the skin. It is very important to have preserved skin pH value after application of topical preparations. pH can influence skin barrier function, thus, its change can disrupt antibacterial protection and homeostasis of the skin [51].

Another good indicator of skin barrier function is EI. Erythema occurs after skin has been injured or exposed to irritants [52]. As can be seen in Figure 4, none of the investigated preparations caused significant changes of EI, and consequently did not cause irritation of the skin.

Even though all of the tested preparations were marked as preparations with good sensory characteristics (easy to spread, not sticky, not greasy, leaving slightly shiny skin), there were some differences between emulgels and creams. In comparison to creams, more participants described emulgels as easy to spread, not sticky, and greaseless after application, but with a slower absorption rate, which is consistent with the advantages of emulgels described in the literature [16]. The feeling of a slower absorption rate among participants may be connected to the controlled release of active substances that is characteristic for emulgels, as a dual control release system [53].

## 5. Conclusions

The study confirmed positive effects of emulgel and cream with immortelle extract and hemp oil on the skin. Application of the preparations led to a significant increase in skin hydration and decrease in TEWL, which implied improvement of epidermal barrier function. Moreover, investigated preparations were well tolerated, which was confirmed by the absence of significant change of EI and preserved pH values of the skin within the appropriate range. Bearing this in mind, the potential use of this preparation in conditions accompanied by dry skin may be investigated in the future. Further, this study showed the importance of the appropriate selection of the carrier, and the advantage of using emulgels over creams for active substance delivery, being in accordance with the growing use of emulgels in dermopharmacy and cosmetology.

## Figures and Tables

**Table 1:** Qualitative and quantitative compositions (%, (*w/w*)) of cream samples: active cream (AC) and placebo cream (PAC).

Component	AC	PAC	Function
Oil phase			
Caprylic/capric triglycerides	5.50	5.50	emollient
Glyceryl stearate (and) ceteareth-20 (and) ceteareth-12 (and) cetearyl alcohol (and) cetyl palmitate	8.50	8.50	mixed O/W emulsifier
Cetyl alcohol	0.75	0.75	emollient
Stearyl alcohol	0.75	0.75	emollient
Water phase			
Glycerin	2.00	2.00	humectant
Sodium benzoate	0.50	0.50	preservative
Distilled water to	100.0	100.0	water phase
Active substances of plant origin			
Hemp (*Cannabis sativa*) oil	1.50	-	active component
Immortelle (*Helichrysum italicum*) extract	1.00	-	active component

**Table 2:** Qualitative and quantitative compositions (%, (*w/w*)) of emulgel samples: active emulgel (EM) and placebo emulgel (PEM).

Component	EM	PEM	Function
Oil phase			
Caprylic/capric triglyceridez	3.50	3.50	emollient
Polysorbate 80	1.50	1.50	O/W emulsifier
Butyrospermum parkii	1.50	1.50	emollient
Tocopheryl acetate	0.50	0.50	antioxidant
BHT	0.01	0.01	antioxidant
Water phase			
Carbomer	1.00	1.00	gelling agent
Propylene glycol	5.00	5.00	humectant
Sodium benzoate	0.50	0.50	preservative
Sodium hydroxide	3.00	3.00	neutralizing agent
Distilled water to	100.0	100.0	water phase
Active substances of plant origin			
Hemp (*Cannabis sativa*) oil	1.50	-	active component
Immortelle (*Helichrysum italicum*) extract	1.00	-	active component

**Table 3:** Sensory evaluation questionnaire.

Before Application	
Consistency	liquid/semi-solid
Gloss level	matte/pearl gloss/slightly gloss/gloss/very gloss
**During Application**	
Spreadability	easy to spread/difficult to spread/very difficult to spread
Adhesion	not sticky/slightly sticky/sticky/very sticky
Density	rare/slightly dense/dense/very dense
Grease	not greasy/slightly greasy/greasy/very greasy
Gloss	not shiny/slightly shiny/shiny/very shiny
Absorption rate	slow/moderate/fast
**After Application**	
Residual film	no film/moderate film/expressive film
Stickiness	not sticky/slightly sticky/sticky/very sticky
Grease	not greasy/slightly greasy/greasy/very greasy
Gloss	not shiny/slightly shiny/shiny/very shiny

**Table 4:** GC profile of fatty acids methyl esters of hemp oil.

No	Name	CAS	KI	% *
1.	Methyl tetradecanoate	124-10-7	1722	0.09
2.	*n*-hexadecanol	36653-82-4	1874	0.12
3.	methyl palmitate (methyl hexadecanoate)	112-39-0	1921	6.27
4.	γ-linolenic acid, methyl ester (6Z,9Z,12Z-octadecatrienoic acid, methyl ester, ω-6)	506-26-3	2091	2.46
5.	methyl steridonate (6Z,9Z,12Z,15Z-octadecatetraenoic acid, methyl ester, ω-3)	73097-00-4	2088	0.99
6.	linoleic acid methyl ester (9Z,12Z-octadecadienoic acid, methyl ester, ω-6)	112-63-0	2095	58.01
7.	methyl oleate (9Z-octadecenoic acid, methyl ester, ω-9)	112-62-9	2108	12.89
8.	α-linolenic acid, methyl ester (9Z,12Z,15Z-octadecatrienoic acid, methyl ester, ω-3)	301-00-8	2108	15.12
9.	methyl stearate (octadecanoic acid, methyl ester)	112-61-8	2123	2.42
10.	methyl-12-hydroxy-9-octadecanoate	141-24-2	2247	0.23
11.	11Z-eicosenoic acid, methyl ester (ω-9)	02.09.2390	2302	0.32
12.	arachidic acid, methyl ester	1120-29-1	2339	0.63
13.	behenic acid, methyl ester (docosanoic acid, methyl ester)	929-77-1	2527	0.22
14.	lignoceric acid, methyl ester (tetracosanoic acid, methyl ester)	2442-49-1	2729	0.08

* Percentage area.

**Table 5:** The results of sensory analysis of the investigated samples.

Before Application							
	Consistency	Gloss Level					
AC	Semisolid (84.6%)	Matte (61.5%)					
PAC	Semisolid (92.3%)	Slightly gloss (46.2%)					
EM	Semisolid (61.5%)	Pearl gloss (53.8%)					
PEM	Semisolid (69.2%)	Pearl gloss (38.5%)					
**During Application**							
	Spreadability	Adhesion		Density	Grease	Gloss	Absorption rate
AC	Easy to spread (100%)	Not sticky (61.5%)		Slightly dense (69.2%)	Not greasy (50%)	Slightly shiny (50%)	Fast (50%)
PAC	Easy to spread (76.9%)	Not sticky (46.2%)		Dense (46.2%)	Not greasy (53.8%)	Slightly shiny (53.8%)	Fast (50%)
EM	Easy to spread (92.3%)	Not sticky (69.2%)		Rare (46.2%)	Not greasy (46.2%)	Slightly shiny (69.2%)	Moderate (69.2%)
PEM	Easy to spread (100%)	Not sticky (53.8%)		Rare (53.8%)	Not greasy (69.2%)	Slightly shiny (53.8%)	Moderate (61.5%)
**After Application**							
	Residual film	Stickiness	Grease		Gloss		
AC	Moderate film (69.2%)	Slightly sticky (53.8%)	Not greasy (46.2%)		Slightly shiny (61.5%)		
PAC	Moderate film (53.8%)	Slightly sticky (76.9%)	Not greasy (46.2%)		Slightly shiny (53.8%)		
EM	Moderate film (61.5%)	Not sticky (100%)	Not greasy (100%)		Slightly shiny (38.5%)		
PEM	Moderate film (76.9%)	Not sticky (61.5%)	Not greasy (53.8%)		Slightly shiny (46.2%)		

The table shows the most common answers with the percentage of respondents who rated the creams (active cream—AC, placebo cream—PAC) and emulgels (active emulgel—EM, placebo emulgel—PEM) in this manner.

## Data Availability

The data presented in this study are available upon request from the corresponding author.
